# Microtubule Dynamics Control HGF-Induced Lung Endothelial Barrier Enhancement

**DOI:** 10.1371/journal.pone.0105912

**Published:** 2014-09-08

**Authors:** Xinyong Tian, Yufeng Tian, Nurgul Moldobaeva, Nicolene Sarich, Anna A. Birukova

**Affiliations:** Department of Medicine, University of Chicago, Chicago, Illinois, United States of America; University of Illinois at Chicago, United States of America

## Abstract

Microtubules (MT) play a vital role in many cellular functions, but their role in peripheral actin cytoskeletal dynamics which is essential for control of endothelial barrier and monolayer integrity is less understood. We have previously described the enhancement of lung endothelial cell (EC) barrier by hepatocyte growth factor (HGF) which was associated with Rac1-mediated remodeling of actin cytoskeleton. This study investigated involvement of MT-dependent mechanisms in the HGF-induced enhancement of EC barrier. HGF-induced Rac1 activation was accompanied by phosphorylation of stathmin, a regulator of MT dynamics. HGF also stimulated MT peripheral growth monitored by time lapse imaging and tracking analysis of EB-1-decorated MT growing tips, and increased the pool of acetylated tubulin. These effects were abolished by EC pretreatment with HGF receptor inhibitor, downregulation of Rac1 pathway, or by expression of a stathmin-S63A phosphorylation deficient mutant. Expression of stathmin-S63A abolished the HGF protective effects against thrombin-induced activation of RhoA cascade, permeability increase, and EC barrier dysfunction. These results demonstrate a novel MT-dependent mechanism of HGF-induced EC barrier regulation via Rac1/PAK1/stathmin-dependent control of MT dynamics.

## Introduction

Enhancement of the endothelial cell (EC) peripheral actin cytoskeleton and increased assembly of cell adhesive complexes by barrier protective agonists provide a structural basis for the maintenance of vascular barrier integrity and prevent catastrophic consequences of uncontrolled vascular leakiness in the lung or other organs caused by bacterial pathogens, cytokine storm accompanying sepsis, trauma, or excessive mechanical forces [1]–[6].

Hepatocyte growth factor (HGF) is a multifunctional mesenchyme-derived pleiotropic factor secreted by several cell types. Along with other bioactive substances it appears in lung circulation under pathological conditions, such as acute lung injury, sepsis, lung inflammation, and ventilator-induced lung injury (VILI), and regulates a number of biological events such as cell mitogenesis, morphogenesis, organogenesis, and cell survival [7]–[9]. In addition, HGF enhances basal EC monolayer barrier properties and exhibits potent protective effects against vascular endothelial barrier compromise induced by agonists and pathologically relevant mechanical forces [10]–[12]. HGF-induced EC barrier protection is mediated by PI3-kinase-dependent activation of Rac-GTPase signaling [13], which causes downregulation of the barrier disruptive RhoA pathway [11]. However, the precise mechanism of HGF-induced negative Rac-Rho crosstalk remains poorly understood.

Increasing evidence suggests the role of microtubule (MT) dynamics in control of EC permeability. MT depolymerization by plant-derived MT poisons vinblastin and nocodazole induces robust activation of Rho signaling leading to Rho-kinase dependent microfilament reorganization, stress fiber formation and actomyosin contraction [14]. MLC phosphorylation observed during MT depolymerization is a result of activated small GTPase Rho and its effector Rho-kinase driven by Rho-specific guanine nucleotide exchange factor GEF-H1 which localizes on microtubules. In MT-bound state, the guanine-exchange activity of GEF-H1 is suppressed, whereas GEF-H1 release caused by MT disassembly stimulates Rho-specific GEF activity [15], [16]. Indeed, partial disassembly of MTs was linked to additional activation of Rho signaling and further enhanced EC barrier dysfunction induced by thrombin, TGFβ1 and TNFα [17]–[20]. This mechanism defines a signaling crosstalk between MT and actin cytoskeleton leading to increased EC permeability. However, MT-actin crosstalk may also contribute to barrier protection and requires MT stabilization. For example, EC pretreatment with MT stabilizing agent taxol attenuated thrombin-induced Rho pathway of EC permeability [21]. In the absence of pharmacologic MT stabilizers, MT growth and stability is precisely regulated by endogenous MT-associated regulatory proteins. Stathmin is one of the key proteins regulating MT dynamics and has been involved in multiple cellular processes, including regulation of cell cycle, cell migration, tumor metastasis and stability of the neuromuscular junction [22]. At the molecular level, unphosphorylated stathmin destabilizes microtubules by reducing the microtubule polymer mass through sequestration of soluble tubulin into an assembly-incompetent T2S complex (two α:β tubulin dimers per molecule of stathmin), and by increasing the switching frequency (catastrophe frequency) from growth to shortening at plus and minus ends by binding directly to the microtubules. Phosphorylation of stathmin on one or more of its four serine residues (S^16^, S^25^, S^38^, and S^63^) reduces its microtubule-destabilizing activity [23] suggesting a cooperative nature of stathmin phosphorylation at different sites in control of its effects on MT depolymerization. In contrast, reduced stathmin phosphorylation promotes vascular endothelial barrier dysfunction in response to barrier disruptive agonists such as thrombin [24].

This study tested the hypothesis that, in contrast to effects of barrier-disruptive agonists, HGF stimulation promotes growth of peripheral MT growth in the vascular endothelial cells. We also examined involvement of microtubule-associated proteins stathmin and GEF-H1 in downregulation of barrier disruptive Rho signaling underlying barrier-protective effects of HGF. We evaluated a role of Rac/PAK-mediated stathmin phosphorylation as a potential mechanism of HGF-induced MT preservation and negative Rac-Rho crosstalk central to HGF-mediated EC barrier protection.

## Materials and Methods

### Cell culture and reagents

Human pulmonary artery endothelial cells (HPAEC) were obtained from Lonza (East Rutherford, NJ). Human HGF was from R&D (Minneapolis, MN). c-Met kinase inhibitor, N-(3-Fluoro-4-(7-methoxy-4-quinolinyl)phenyl)-1-(2-hydroxy-2-methylpropyl)-5-methyl-3-oxo-phenyl-2,3-dihydro-1H-pyrazole carboxamide, was from Millipore (Billerica, MA). Reagents for immunofluorescence were purchased from Molecular Probes (Eugene, OR). Antibodies to phospho-MYPT, GEF-H1, PAK1, phospho-MLC, phospho-Y^421^ cortactin were from Cell Signaling (Beverly, MA); stathmin, and End-Binding protein-1 (EB1) were from BD Transduction Laboratories (San Diego, CA); Rac1, RhoA, His-tag were from Santa Cruz Biotechnology (Santa Cruz, CA). Stathmin phospho-S63 specific antibody, cat. #76583, was from Abcam (Cambridge, MA). Unless otherwise specified, all biochemical reagents were obtained from Sigma (St. Louis, MO).

### Si-RNA and DNA transfections

Pre-designed Rac1-specific siRNA of standard purity was ordered from Ambion (Austin, TX). PAK1-specific set of three Stealth siRNA duplexes was purchased from Invitrogen (Carlsbad, CA). Transfection was performed as previously described [25]. Plasmid encoding stathmin-S63A mutant cloned into pcDNA3.1A vector containing 6xHis-tag and empty vector were kindly provided by G. Bokoch (Scripps, La Jolla, CA) [26]. Transient transfections of HPAEC were carried out using PolyJet reagent from Signagen Laboratories (Rockville, MD) as recommended by the manufacturer [21], [27]. For more effective introduction of cDNA into the cell, nucleofection of HPAEC was performed using a kit from Amaxa Biosystems (Lonza, Allendale, NJ). Optimized protocol of nucleofection was provided by the manufacturer and used with minor modifications described previously [18]. After 16–18 hr of transfection, pulmonary EC were treated with either vehicle or HGF and used for experiments. This post-transfection time was sufficient to detect changes in the HGF-stimulated cells expressing S63A mutant, while under basal conditions differences between the cells expressing stahtmin S63A, empty vector, or non-transfected cells were negligible. Of note, the decrease in MT density was observed in EC monolayers at longer post-transfection times.

### RhoA and GEF-H1 activity assays

Active RhoA was captured using GST-rhotekin beads as previously reported [28]. Active GEF-H1 was affinity precipitated from cell lysates as described [29] using the RhoA(G17A) mutant which cannot bind nucleotide and therefore has high affinity for activated GEFs [30]. Plasmid encoding GST-tagged RhoA(G17A) was a gift from K. Szaszi (St. Michael's Hospital, Toronto, Canada).

### Immunofluorescence and live cell imaging

Endothelial monolayers plated on glass cover slips were subjected to immunofluorescence staining with Texas Red phalloidin to visualize F-actin [18], [27]. For microtubule quantification, cells were fixed with 100% methanol cooled to −20°C, and immunostaining was carried out with β-tubulin or EB1 antibodies [24], [31]. Briefly, after the cell boundaries were outlined, the concentric outline shapes reduced to 70% were applied to the images to mark peripheral (outer 30% of diameter) and central (inner 70% of diameter) regions. The integrated fluorescence density in the peripheral area was measured using MetaMorph software and was calculated as a percentage of the integrated fluorescence density in the total cell area. The results were normalized in each experiment. Similar methods were applied to EB1 quantification in fixed cells except that EB1 immunoactivity was manually counted and results were not normalized. Minimum 10 cells per condition, in three experimental repeats were analyzed. For time lapse microtubule plus end tracking, cells were seeded on MatTek dishes (MatTek, Ashland, MA) and transfected with GFP-EB1. Images were acquired with 100× NA 1.45 oil objective in a 3I Marianas Yokogawa-type Spinning Disk Confocal system equipped with a CO_2_ chamber and a heated stage. Time lapse images were taken with 2 second intervals for 40 to 60 seconds. 20 consecutive images in each condition were used for projection analysis using ImageJ software. For tracking analysis, EB1 in the cell margin area (2–10 µm from cell border) was tracked with the Manual Tracking plug-in in ImageJ software. The median track length was calculated using Excel software.

### Subcellular fractionation

MT-enriched fractions were isolated as previously described [21]. Briefly, cells were incubated with buffer containing PEM (100 mM Pipes pH 6.75, 1 mM EGTA, 1 mM MgSO4, pH 6.75), 0.5% NP-40 (10 min, RT). Cytosolic fraction containing soluble tubulin was collected by centrifugation (12000 rpm, 15 min, RT). The attached cells containing polymerized MT were incubated on ice for 30 min to induce microtubule depolymerization and tubulin release into the soluble fraction. Cells were scraped in PEM; the cell debris was removed by centrifugation (2000 g, 2 min, 4°C). Protein extracts were separated by SDS-PAGE.

### Measurement of transendothelial electrical resistance

Measurements of transendothelial electrical resistance (TER) across HPAEC were performed using the electrical cell-substrate impedance sensing system (ECIS) (Applied Biophysics, Troy, NY) as described [21], [32]. For measurements of TER changes in HGF- or thrombin-stimulated EC expressing stathmin S63A mutant or empty vector control, we used EC nucleofection in suspension followed by cell plating at high density. Permeability assays we performed 16–18 hrs after cell plating.

### Statistical analysis

Results are expressed as means ± SD of three to five independent experiments. Stimulated samples were compared to controls by unpaired Student's *t*-tests. For multiple-group comparisons, a one-way variance analysis (ANOVA), followed by the post hoc Fisher's test, were used. P<0.05 was considered statistically significant.

## Results

### HGF-induced peripheral MT elongation is mediated by Rac1

**Figure 1:**
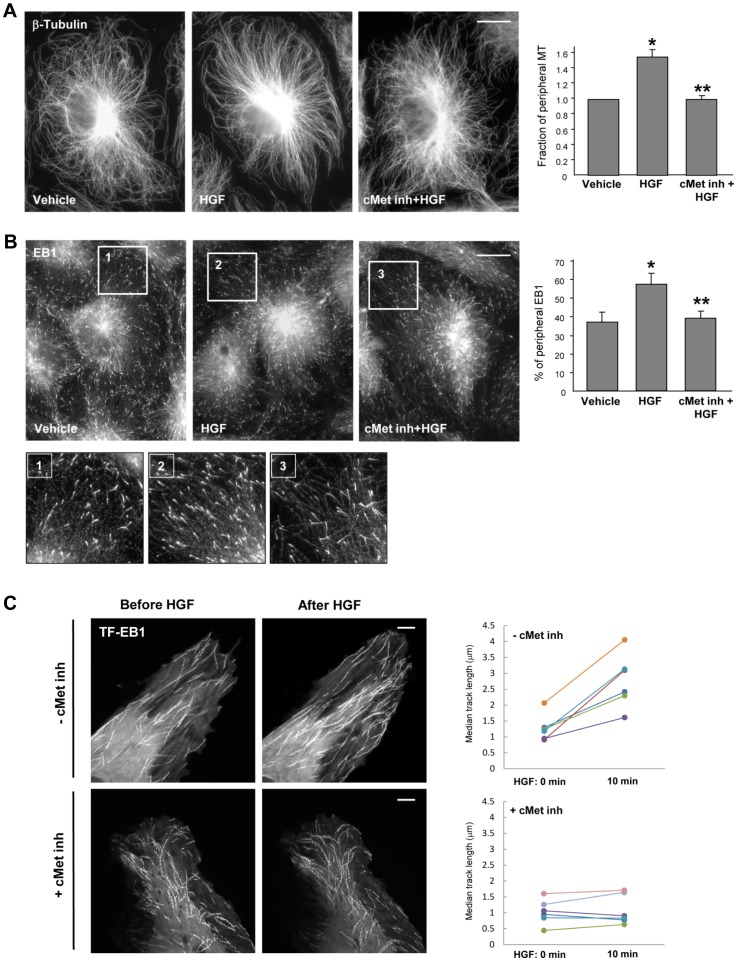
HGF stimulates peripheral MT growth. HPAEC grown on coverslips were stimulated with HGF (50 ng/ml, 10 min) with or without pretreatment with c-Met inhibitor (carboxamide 50 nM, 30 min) followed by **A**: Immunofluorescence staining with an antibody against β-tubulin; **B**: Immunostaining with anti-EB1 antibody. Insets show high magnification images of cell periphery areas with microtubules or EB1-positive microtubule tips. Bar  =  5 µm. Results are representative of five independent experiments. Bar graphs depict results of quantitative analysis of peripheral microtubules (**A, right panel**) and peripheral EB1 (**B, right panel**) in methanol-fixed HPAEC; *P<0.05; n = 4; 6 images from each experiment. **C**: Live cell imaging of HPAEC expressing GFP-EB1 stimulated with HGF with or without pretreatment with c-Met inhibitor. Projection analysis of 20 consecutive images before and after HGF treatment shows changes in GFP-EB1 track length. Bar  =  2 µm. Quantification of GFP-EB1 track length is presented on right panels. Each pair of dots represents the median track length in a cell before and after thrombin treatment. Results are representative of four independent experiments; eight cells have been inspected for each condition, in each experiment.

To assess HGF effects on peripheral MT density and growth, control and HGF-stimulated EC were fixed with methanol and subjected to immunofluorescence staining with antibody to β-tubulin or End-Binding protein-1 (EB1) which tracks the growing plus end of microtubules. Analysis of MT structure showed expanded peripheral microtubule network in HGF-treated EC (**Figure 1A**) and higher density of EB1-positive MT tips at the cell distal area, as shown in **Figure 1B** and higher magnification insets. HGF effects on peripheral MT expansion were abolished by cell pretreatment with c-Met inhibitor carboxamide. Quantitative analysis of immunofluorescence data is presented in **Figure 1A and B, right panel** **s**.

Effects of HGF on microtubule dynamics were further examined using a live imaging approach. For this purpose, EC were transfected with GFP-tagged EB1 which tracks the growing plus end of microtubules. EB1 tracking in live cells was performed by live videomicroscopy, and projection images were generated as described in the Methods section. HGF stimulation increased the length of EB1 tracks, which represents episodes of uninterrupted microtubule growth (**Figure 1D** **, top panel**). These HGF effects were abolished by cell pretreatment with the c-Met inhibitor (**Figure 1D** **, top panel**).

**Figure 2:**
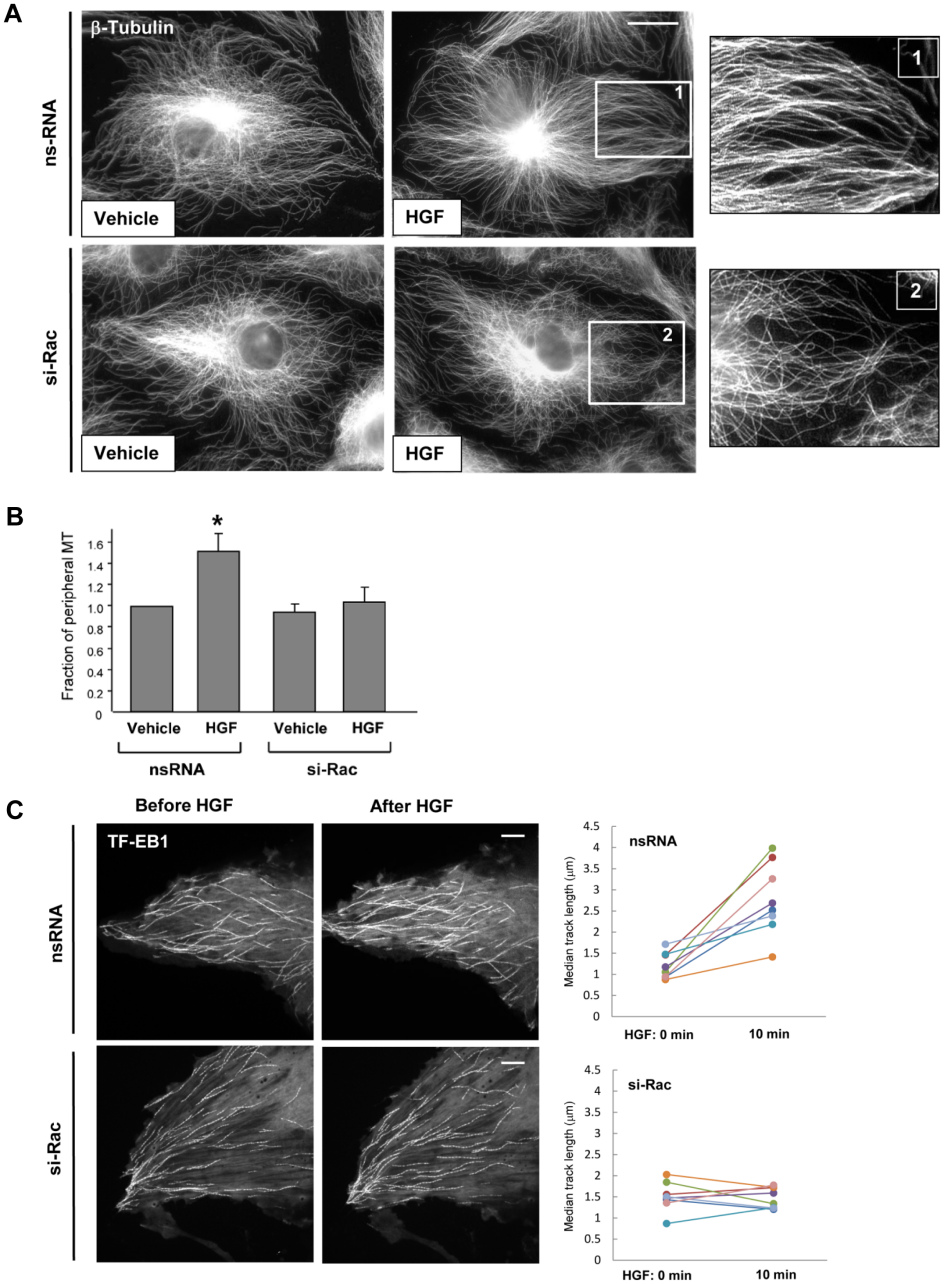
Role of Rac1 in HGF-induced stimulation of peripheral MT network formation. **A**: Cells grown on coverslips were transfected with non-specific RNA or Rac1-specific siRNA and stimulated with HGF (50 ng/ml, 10 min) followed by immunofluorescence staining with an antibody against β-tubulin. Bar  =  5 µm. Magnified images (insets) show details of MT structure. Results are representative of four independent experiments. **B**: Fraction of peripheral MT was quantified as described in Methods; *P<0.05; n = 4; 6 images from each experiment. **C**: Projection analysis of 20 consecutive images in control (**top panel**) and Rac1 knockdown (**bottom panel**) live cells before and after HGF treatment shows changes in GFP-EB1 track length. Bar  =  2 µm. Quantification of GFP-EB1 track length is presented on right panels. Results are representative of four independent experiments; eight cells have been inspected for each condition, in each experiment.

The role of Rac mechanism in HGF-induced effects on MT growth was tested in experiments with siRNA-induced Rac1 knockdown. Rac1 depletion attenuated the HGF-induced increase in the density of peripheral microtubules visualized by immunofluorescence staining of β-tubulin in methanol-fixed EC monolayers (**Figure 2A**). Quantitative analysis of the peripheral MT pool is presented in **Figure 2B**. A complementary approach using live imaging of GFP-EB1-decorated MT growing plus ends also showed attenuation of MT continuous growth in HGF-stimulated EC with depleted Rac1 (**Figure 2C**). Collectively, these results demonstrate that HGF promotes both the length of continuous microtubule growth and the number of growing ends at the cell margin in a c-Met and Rac1-dependent fashion.

### HGF induces stathmin phosphorylation and increases the pool of stable MT by a Rac1-dependent mechanism

Stathmin phosphorylation at S^63^ increases microtubule stabilization, which is important for the EC barrier maintenance [24]. We next tested whether HGF increases stathmin phosphorylation and if this process is mediated by a cMet – Rac1 – PAK1 dependent mechanism.

**Figure 3:**
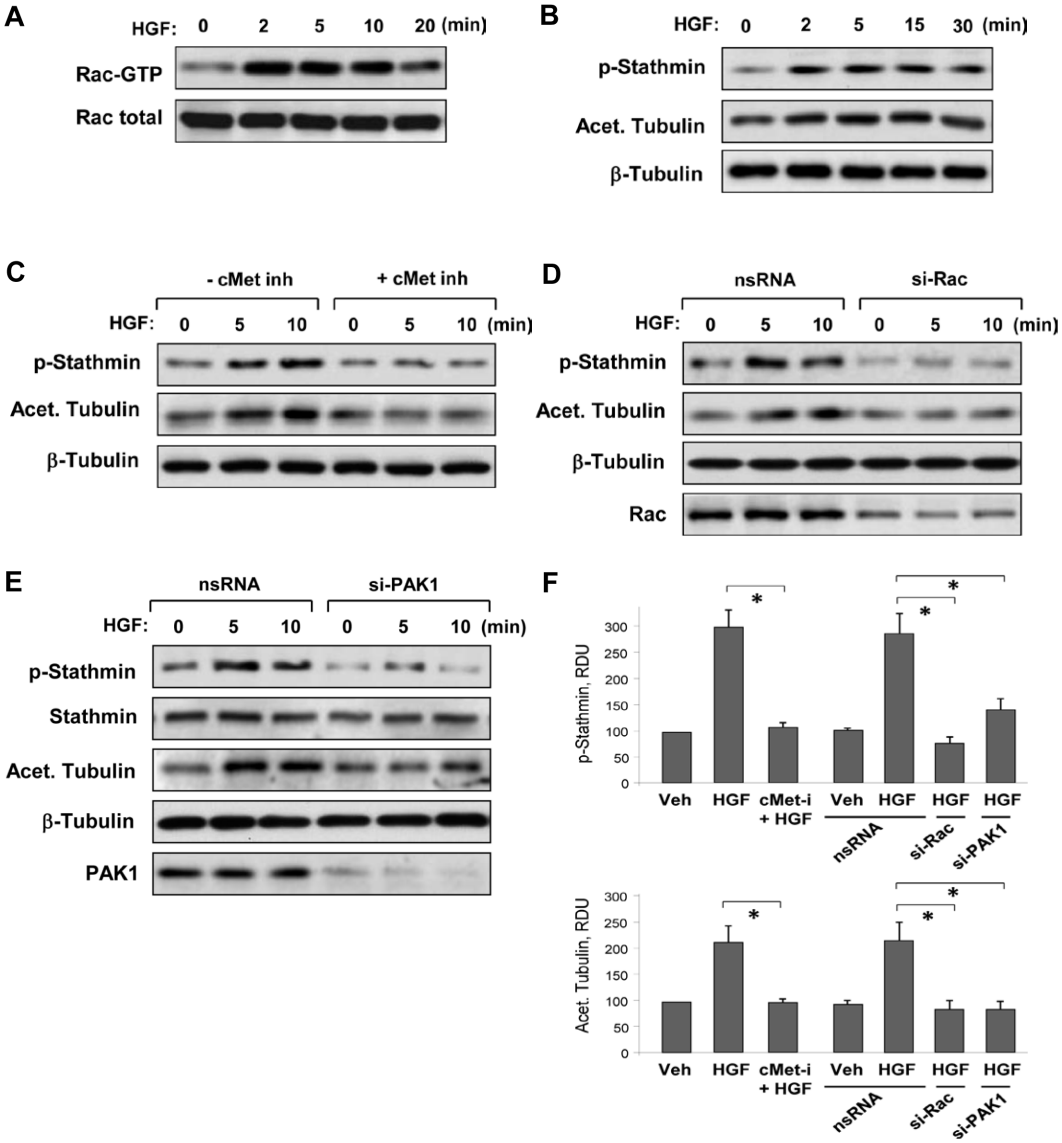
Involvement of Rac pathway in HGF-induced MT-associated signaling. **A–D**: HPAEC were subjected to pretreatment with c-Met inhibitor (carboxamide 50 nM, 30 min) or knockdown of Rac1 or PAK1 as described in Methods and stimulated with HGF (50 ng/ml) for the indicated periods of time. **A**: Rac activation was determined by Rac-GTP pulldown assay. The content of activated Rac was normalized to the total Rac content in EC lysates. **B**: Time-dependent stimulation of stathmin phosphorylation and increase in tubulin acetylation was detected by western blot. **C**: Effect of preincubation with c-Met inhibitor on HGF-induced stathmin phosphorylation was evaluated by western blot with phospho-S^63^-stathmin antibody. **D and E**: HGF-induced stathmin phosphorylation and tubulin acetylation in cells with Rac1 (**D**) and PAK1 (**E**) knockdown were evaluated by western blot. siRNA-induced target protein depletion was confirmed by membrane probing with Rac1 or PAK1 antibody. Equal protein loading in all assays was confirmed by membrane probing with β-actin antibody. **F**: Bar graphs depict the quantitative densitometry analysis of western blot data from four independent experiments; *P <0.05, RDU: relative density units.

HGF treatment rapidly stimulated Rac1 within 2 min (**Figure 3A**) and increased stathmin phosphorylation (**Figure 3BF**). These events were also accompanied by an increase in acetylated tubulin. HGF-induced stathmin phosphorylation and increase in acetylated tubulin were abolished by cell pretreatment with c-Met inhibitor (**Figure 3CF**) or by si-RNA-induced knockdown of Rac1 (**Figure 3DF**). Involvement of PAK1 kinase in the HGF-induced stathmin phosphorylation was directly tested in experiments with siRNA-induced PAK1 knockdown (**Figure 3EF**). The results showed that depletion of endogenous PAK1 abolished both HGF-induced stathmin phosphorylation and the increase in the pool of acetylated tubulin. These results demonstrate the role of the c-Met – Rac1 – PAK1 axis in the HGF-induced modulation of stathmin activity and MT dynamics.

### Stathmin phosphorylation mediates HGF-induced endothelial barrier enhancement, actin remodeling and increased MT peripheral growth

**Figure 4:**
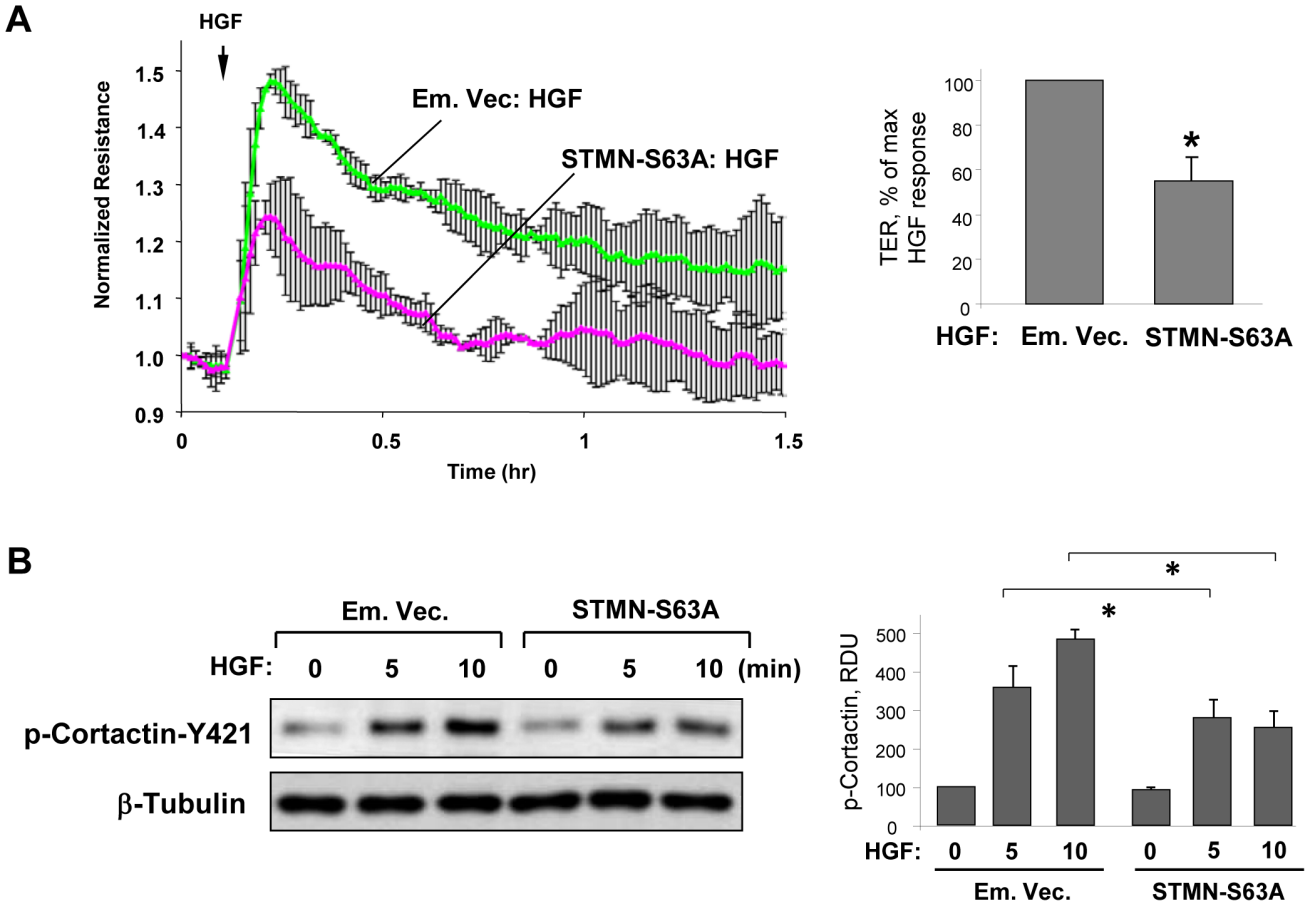
Expression of phosphorylation-deficient stathmin attenuates HGF-induced EC barrier enhancement. **A**: Endothelial monolayers transfected with phosphorylation-deficient stathmin (STMN-S63A) or empty vector (Em. Vec.) were stimulated with HGF (50 ng/ml). **A**: TER measurements were performed over 1.5 hrs. Bar graphs depict results of quantitative analysis of permeability data; n = 5; *P<0.05. **B**: Cortactin phosphorylation at Y^421^ and tubulin acetylation at indicated time points of HGF treatment was monitored by Western blot. Probing for β-tubulin was used as a normalization control. Results are representative of three independent experiments. Bar graphs depict the quantitative densitometry analysis of western blot data; n = 4; *P <0.05, RDU: relative density units.

The role of stathmin phosphorylation in HGF-induced EC barrier enhancement and associated activation of cortical actin cytoskeleton was further tested in experiments with a phosphorylation deficient stathmin mutant, stathmin-S63A. Ectopic expression of stathmin-S63A attenuated HGF-induced increase in transendothelial electrical resistance, which reflects enhancement of EC barrier (**Figure 4A**). Interestingly, expression of stathmin-S63A also decreased HGF-induced phosphorylation of cortactin at Y^421^ (**Figure 4B**). Rac activation triggers signaling pathways leading to increased cortactin tyrosine phosphorylation. Cortactin tyrosine phosphorylation may be stimulated by increased Rac1 activity [33] leading to activation of cortactin peripheral translocation and peripheral actin cytoskeletal dynamics [34]. Altogether, these data suggest an additional mechanism of cortical cytoskeletal regulation by HGF via stathmin phosphorylation.

**Figure 5:**
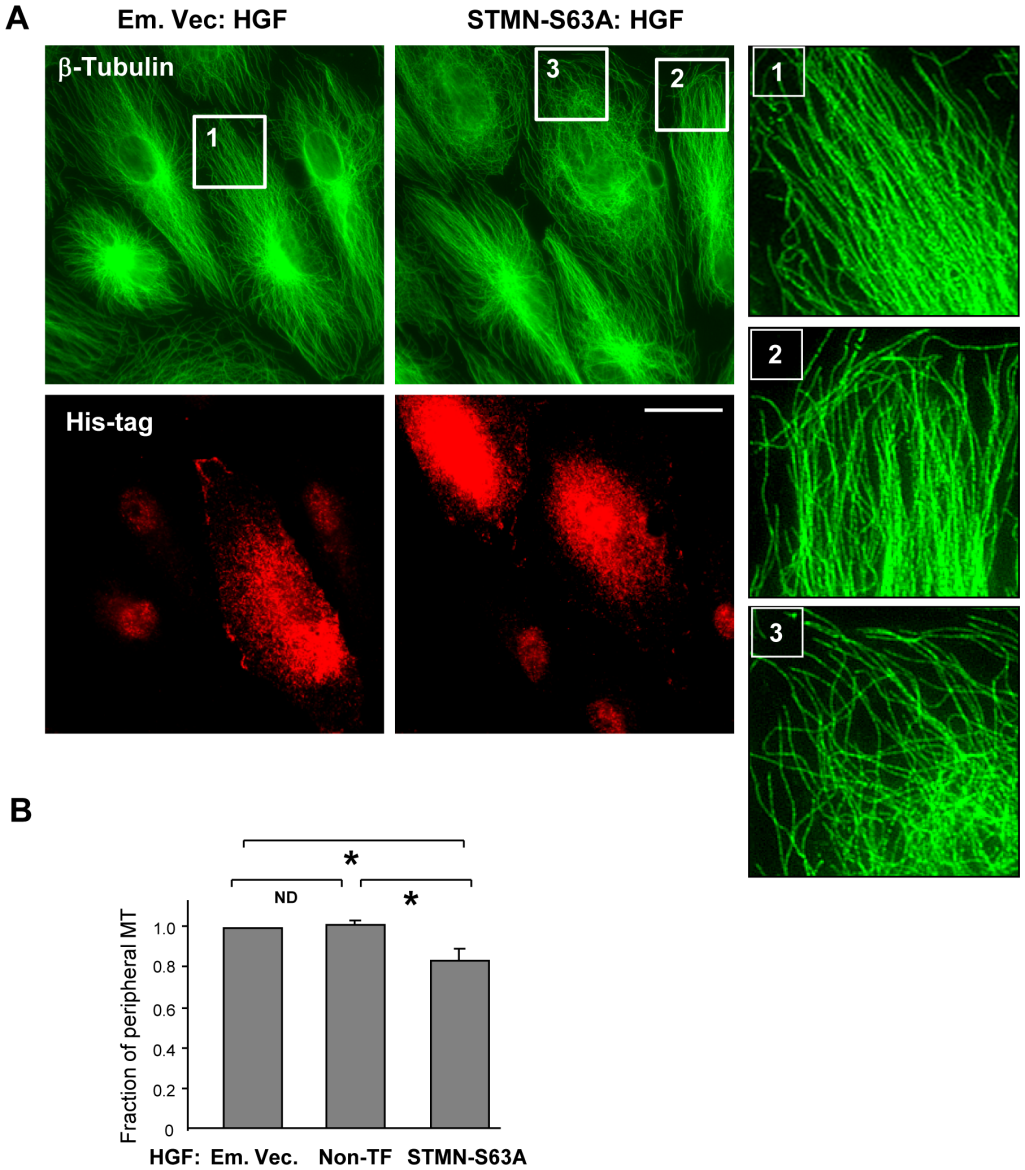
Expression of phosphorylation-deficient stathmin attenuates HGF-induced stimulation of peripheral MT network formation. Cells grown on coverslips were transfected with empty vector (Em. Vec.) or STMN-S63A and stimulated with HGF (50 ng/ml, 10 min). A: MT network was visualized by immunofluorescence staining of methanol-fixed cells with an antibody against β-tubulin. Transfected cells were detected by staining with His-tag antibody. Insets show magnified images with details of MT structure in non-transfected and STMN-S63A transfected cells. Bar  =  10 µm. Results are representative of three independent experiments. B: Bar graphs depict results of quantitative analysis of peripheral microtubules; n = 3; 10 cells from each experiment; *P<0.05.

Because phosphorylation of stathmin on one or more of its four serine residues (S^16^, S^25^, S^38^, and S^63^) reduces its microtubule-destabilizing activity [23], we next evaluated the role of stathmin phosphorylation at S^63^ in HGF-induced MT growth. To minimize adverse effects of stathmin-S63A overexpression on global MT arrangement, we used the EC cultures at earlier times after transfection with stathmin-S63A plasmid (16–18 hrs). This post-transfection time was sufficient to detect the changes in the HGF-stimulated cells expressing S63A mutant (**Figure 5**), while under basal conditions the differences between the cells expressing stahtmin S63A, empty vector, or non-transfected cells were negligible. The results of immunofluorescence staining of methanol-fixed EC show that expression of stathmin-S63A decreases the density of peripheral microtubules in HGF-stimulated EC (**Figure 5**).

### Stathmin phosphorylation is involved in HGF inhibitory effects on thrombin-induced endothelial barrier dysfunction

Agonist-induced activation of RhoA leading to increased EC permeability may be modulated by the microtubule cytoskeleton via association-dissociation balance of MT-bound Rho specific nucleotide exchange factor, GEF-H1 [35], [36]. Because peripheral MT dynamics are intimately involved in the mechanisms of MT-actin signaling crosstalk, the effects of stathmin-S63A expression on agonist-induced MT remodeling and Rho signaling were further investigated.

**Figure 6:**
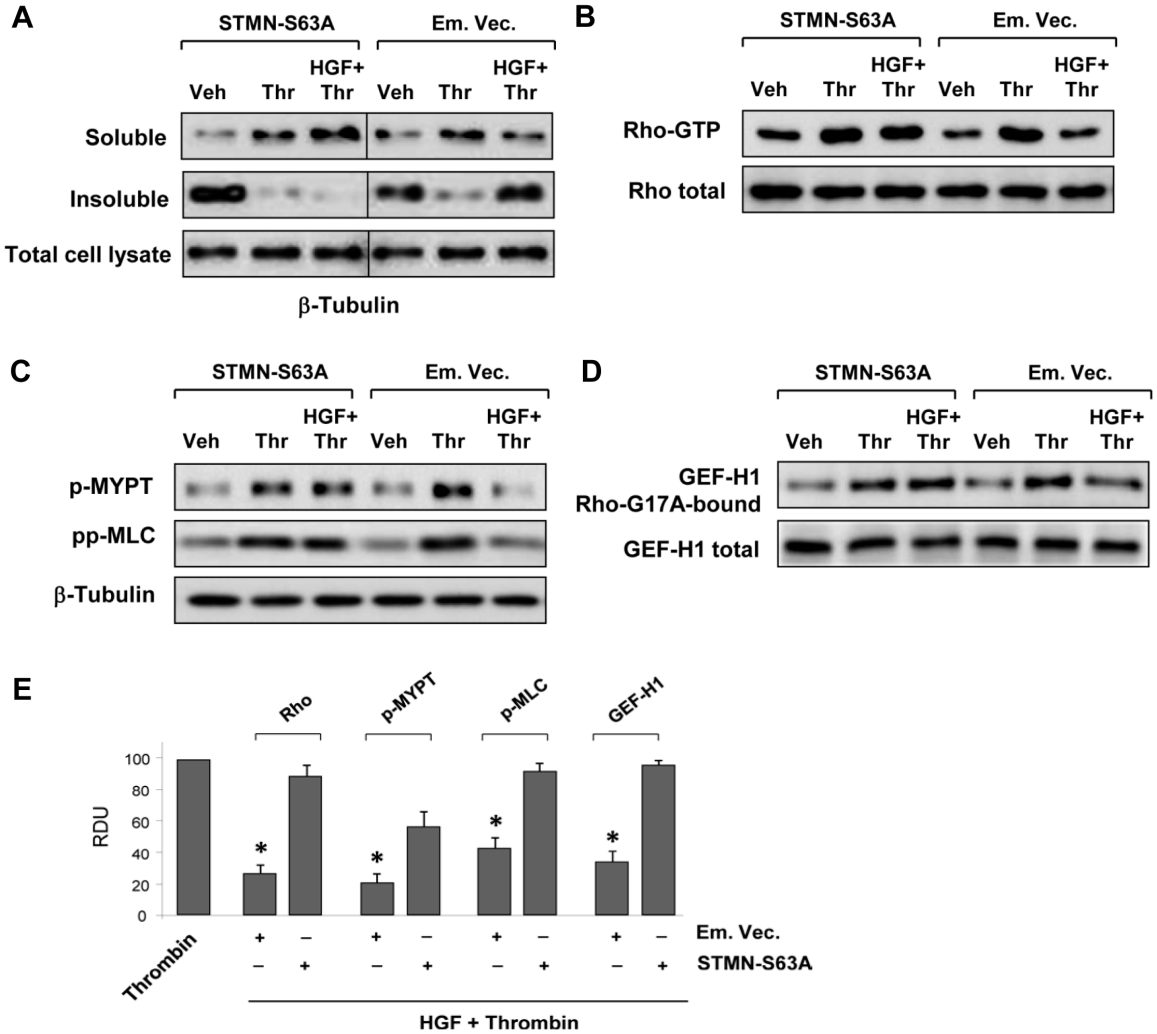
Phosphorylation-deficient stathmin attenuates HGF protective effects against thrombin-induced MT disassembly and activation of Rho signaling. Cells transfected with empty vector (Em. Vec.) or STMN-S63A were stimulated with thrombin (0.5 U/ml, 15 min) or HGF (50 ng/ml, 10 min) + thrombin. A: After separation of MT-enriched fraction and fraction with soluble β-tubulin, β-tubulin content in each fraction was determined by western blot. Determination of β-tubulin content in total cell lysates was used as a normalization control. B: Rho activity in total cell lysates was evaluated by RhoGTP pulldown assay (upper panel) and normalized to total Rho content in cell lysates (middle panel). C: Activation of the Rho pathway was evaluated by western blot analysis of phospho-MYPT and diphospho-MLC levels. Reprobing with β-actin antibody was used as the normalization control. D: GEF-H1 activation in HPAEC stimulated with thrombin or HGF + thrombin was evaluated by GEF pulldown assay. Western blot detection of GEF-H1 in corresponding total lysates was used as normalization control. E: Bar graphs depict the quantitative densitometry analysis of western blot data from three independent experiments; *P <0.05, RDU: relative density units.

Subcellular fractionation experiments showed that thrombin stimulation decreased the pool of insoluble tubulin with a concomitant increase in the soluble fraction which reflects thrombin-induced partial MT depolymerization. Thrombin-induced MT depolymerization was attenuated by cell co-treatment with HGF. Expression of stathmin-S63A abolished HGF's inhibitory effect on thrombin-induced MT depolymerization (**Figure 6A**). Stathmin-S63A expression also impaired the inhibitory effect of HGF on thrombin-induced Rho activation (**Figure 6BE**) and Rho-kinase mediated phosphorylation of myosin-associated phosphatase (MYPT) and myosin light chain (MLC) (**Figure 6CE**) induced by thrombin. Direct analysis of GEF-H1 activity showed that thrombin-induced GEF-H1 activation was attenuated by HGF. Expression of stathmin-S63A abolished HGF-mediated downregulation of GEF-H1 activity (**Figure 6DE**).

**Figure 7:**
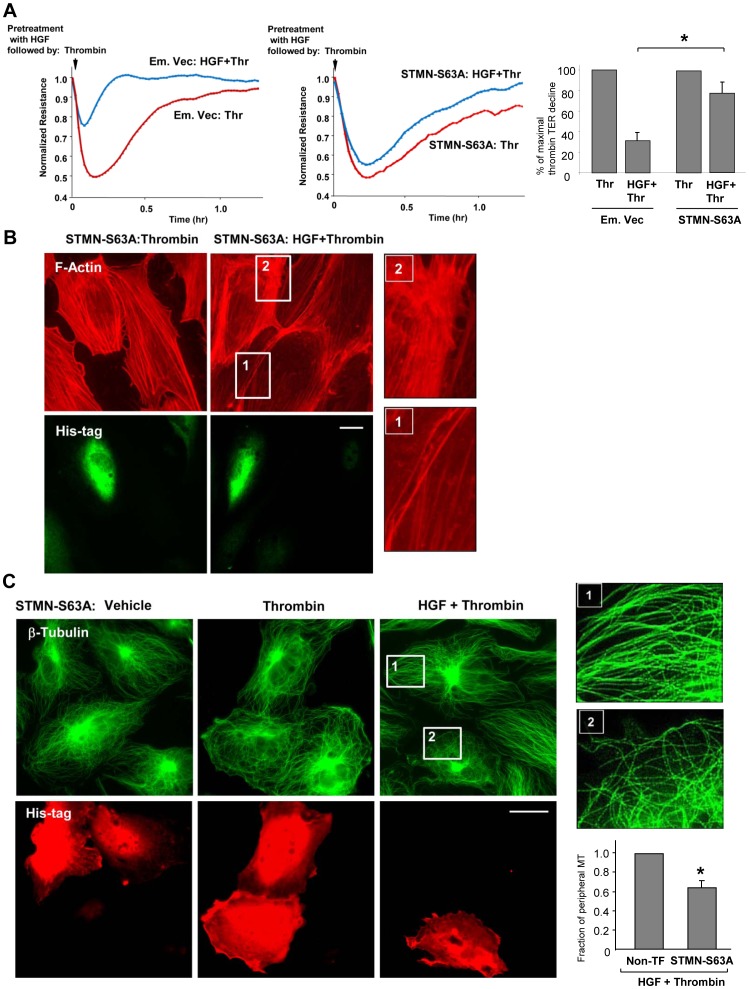
Phosphorylation-deficient stathmin attenuates HGF protective effects against thrombin-induced permeability and cytoskeletal remodeling. Endothelial monolayers transfected with phosphorylation-deficient stathmin (STMN-S63A) or empty vector (Em. Vec.) were treated with thrombin or HGF (50 ng/ml) + thrombin (0.5 U/ml). A: TER measurements were performed over 1.5 hrs. Results are representative of four independent experiments. Bar graphs depict results of quantitative analysis of TER data; *P<0.05. B-C: F-actin remodeling (B, bar  =  5 µm) and MT remodeling (C, bar  =  10 µm) in EC expressing STMN-S63A was performed by double immunofluorescence staining with either Texas Red phalloidin, or β-tubulin antibody and His-tag antibody to detect STMN-S63A expressing cells. The magnified images (insets) show the details of actin and MT structure in non-transfected and STMN-S63A expressing cells. Results are representative of three independent experiments. Bar graphs (C, lower right panel) depict results of quantitative image analysis of peripheral microtubules from three independent experiments; 10 cells from each experiment. *P<0.05.

Impaired downregulation of Rho signaling in HGF-treated cells with stathmin-S63A expression suggests significant functional consequences related to mechanisms of HGF-induced vascular endothelial barrier recovery. We examined effects of stathmin-S63A on thrombin-induced EC permeability and protective effects of HGF. Measurements of TER changes in response to thrombin and HGF were performed in EC monolayers transfected with the stathmin-S63A mutant or the control vector. Transfection with stathmin-S63A significantly reduced the barrier protective effect of HGF in thrombin-stimulated EC monolayers (**Figure 7A**). In contrast to attenuation of thrombin effects by HGF observed in non-transfected cells or cells transfected with empty vector (**Figure 7B**), expression of stathmin-S63A attenuated HGF-induced inhibition of thrombin-induced stress fiber formation.

We next examined effects of stathmin-S63A expression on microtubule changes in response to EC stimulation with thrombin or combined thrombin and HGF treatment. Expression of stathmin-S63A did not considerably affect the MT structure or cell morphology in basal conditions or upon thrombin stimulation (**Figure 7C**). However, after combined treatment with thrombin and HGF, the cells with stathmin-S63A expression revealed much lower peripheral MT density in comparison to their non-transfected neighbors.

## Discussion

The results of this study show for the first time that barrier enhancing effects of HGF on vascular endothelium are associated with increased peripheral MT growth. Increased endothelial permeability induced by several proinflammatory agonists including thrombin, TGFβ and TNFα is accompanied by partial dissolution of peripheral microtubules, while MT stabilization by taxol partially attenuated the agonist-induced EC permeability response [19], [21]. It is, however, unclear whether effects of barrier enhancing agonists are associated with the opposite MT response, i.e. increased density of peripheral MT. Using EB-1 as a tracker of growing microtubule plus ends, we directly analyzed the agonist-induced changes in microtubule dynamics in living endothelial cells. Measurements of mean length values of growing EB-1 positive ends showed a marked increase in MT growth in HGF-stimulated cells which was dependent on c-Met receptor activation, Rac1 and PAK1 signaling. A recent study showed that HGF increased MT growth rate during epithelial remodeling in a physiologically relevant three-dimensional environment [37]. Interestingly, large numbers of MTs grew independently of centrosome reorientation into HGF-induced cell extensions and preceded further morphological changes dependent on actin cytoskeleton [37]. Similarly, MT extensions in lamellopodia-like formations were observed in HGF-stimulated pulmonary EC in our studies (data not shown). These observations suggest the HGF-induced activation of MT - actin crosstalk which requires further investigation and may play an important role in cell migration in 3D environment, restoration of cell monolayer integrity and other cell functions.

Previous studies by the Wadsworth group have shown an increased MT turnover at periphery of HGF-treated polarized epithelial cells [38] as result of increased microtubule growth and shortening average rates in lamellar regions [39]. These effects may explain in part the increased peripheral MT density in the HGF-stimulated pulmonary EC observed in this study. However, our results suggest an additional mechanism of HGF-induced peripheral MT stabilization via Rac-PAK1-mediated phosphorylation and inactivation of the negative regulator of MT assembly stathmin. We have previously shown that stathmin knockdown was protective against thrombin-induced activation of Rho signaling and EC permeability [24]. In this study, we tested effects of modulation of stathmin functional activity by HGF-induced signaling pathways. HGF induced stathmin phosphorylation at S^63^ in stimulated EC was inhibited by pharmacologic HGF receptor inhibitor. Importantly, knockdown of Rac downstream effector kinase PAK1 suppressed stathmin phosphorylation in response to HGF, suggesting the PAK1-dependent mechanism of alteration of microtubule dynamics by HGF. In turn, expression of phosphorylation deficient stathmin-S63A mutant attenuated HGF-induced MT growth, activation of actin remodeling monitored by cortactin phosphorylation, and EC barrier enhancement.

On the other hand, a stathmin mutation at S^63^ was sufficient for attenuation of the HGF effect on thrombin-induced Rho signaling and EC permeability. Rho-specific guanine nucleotide exchange (GEF) activity of MT associated Rho regulator GEF-H1 has been implicated in the regulation of Rho signaling by microtubules. In the microtubule-bound state, the GEF activity of GEF-H1 is suppressed, whereas GEF-H1 release caused by microtubule disruption stimulates its Rho-specific GEF activity [16]. Attenuation of HGF inhibitory effects on GEF-H1 and Rho signaling in EC expressing stathmin-S63A observed in this study are consistent with MT destabilization, and an increased pool of MT-free activated GEF-H1.

Interestingly, despite the existence of multiple stathmin phosphorylation sites (at least four) regulating stathmin interaction with tubulin, the mutation of a single site was sufficient for attenuation of HGF-induced MT growth, effects on Rho signaling and EC permeability. These data suggest an allosteric mechanism of stathmin activity regulation by phosphorylation. An interesting implication of this observation is that MT stability may be regulated via stathmin phosphorylation by different signaling kinases which in turn may be stimulated by different barrier-protective agonists. Our data show that non-phosphorylatable stathmin-S63A mutant attenuates, but not completely blocks, the HGF-induced MT peripheral growth, the HGF-induced EC barrier enhancement response and the HGF-induced suppression of thrombin-induced Rho signaling. This result does not exclude a potential involvement of other MT-associated proteins regulating MT growth and stability (i.e. tau, EB-1) in control of EC permeability. We speculate that the regulation of EC barrier by MT dynamics in the native environment may be a result of the superposition of the effects of these proteins on MT stability and peripheral growth, which ultimately contributes to the EC functional response to agonist stimulation.

HGF-induced stathmin phosphorylation had a profound effect on increased EC barrier properties monitored by TER measurements. Activation of Rac1 results in activation of cytoskeletal effector proteins including cortactin, which triggers peripheral actin polymerization and the formation of the peripheral actin rim [34], [40] essential for EC barrier enhancement. The inhibition of HGF-induced cortactin phosphorylation in cells expressing the stathmin-S63A mutant observed in this study was unexpected and suggests a signaling loop between Rac1-PAK1-mediated phosphorylation of stathmin and stathmin-dependent stimulation of cortactin tyrosine phosphorylation. Cortactin phosphorylation may be regulated by Rac1 indirectly via Rac1-dependent inactivation of submembrane-located LMW tyrosine phosphatase leading to accumulation of tyrosine phosphorylated proteins [41], [42]. The mechanisms of this crosstalk require further investigation.

**Figure 8:**
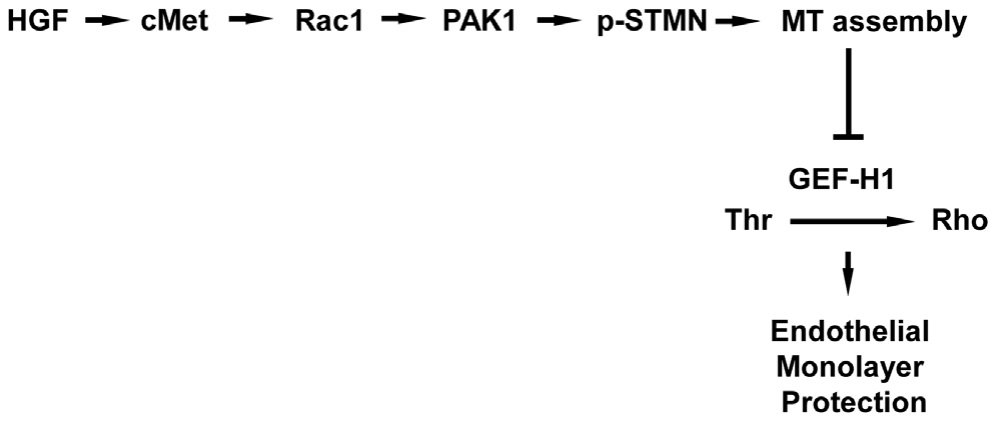
Signaling mechanism of HGF-induced attenuation of GEF-H1 activity and barrier disruptive Rho signaling via Rac1-dependent stimulation of peripheral microtubule network. HGF engages c-Met receptor and triggers Rac1 activity, leading to activation of PAK1. PAK1-mediated phosphorylation of stathmin inhibits its MT destabilizing activity and promotes the growth of the peripheral MT network leading to immobilization and inactivation of Rho-specific guanine nucleotide exchange factor GEF-H1. As a result, MT-dependent GEF-H1 inactivation attenuates agonist induced Rho signaling leading to recovery of peripheral actin cytoskeleton and restoration of endothelial barrier.

In summary, these results provide a functional link between microtubule dynamics and endothelial barrier enhancement by HGF. Based on previous reports and the results of this study, we propose a scheme of HGF-induced small GTPase regulation and vascular endothelial barrier protection (**Figure 8**). HGF binds c-Met receptor [43]–[45] and triggers Rac1 activity, leading to activation of PAK1 and PAK1-mediated phosphorylation of stathmin. Phosphorylated stathmin promotes the growth of the peripheral MT network leading to binding to MT and inactivation of Rho-specific guanine nucleotide exchange factor GEF-H1. As a result, HGF-induced promotion of the MT network leads to the downregulation of Rho signaling, activation of peripheral actin polymerization, cytoskeletal remodeling and endothelial barrier recovery. These findings provide novel mechanistic insights into control of vascular endothelial permeability by HGF and may reflect fundamental mechanisms of EC permeability control by a spectrum of agonists via MT-dependent regulation of Rac-Rho crosstalk.
